# The Negative Effects of High Rates of Biochar on Violas Can Be Counteracted with Fertilizer

**DOI:** 10.3390/plants11040491

**Published:** 2022-02-11

**Authors:** Abishkar Regmi, Sukhbir Singh, Naima Moustaid-Moussa, Cade Coldren, Catherine Simpson

**Affiliations:** 1 Department of Plant and Soil Science, Texas Tech University, Lubbock, TX 79409, USA; abishkar.regmi@ttu.edu (A.R.); s.singh@ttu.edu (S.S.); cade.coldren@ttu.edu (C.C.); 2 Department of Nutritional Sciences, Texas Tech University, Lubbock, TX 79409, USA; naima.moustaid-moussa@ttu.edu; 3 Obesity Research Institute, Texas Tech University, Lubbock, TX 79409, USA

**Keywords:** biochar, Viola, ornamental, physiology, containers, production

## Abstract

Increasing costs and environmental issues regarding excessive use of peat moss is impacting the horticultural industry. Biochar is a valuable substrate additive that has the potential to reduce the use of peatmoss in greenhouse production. However, its varying effects on ornamentals requires that individual species and cultivars of crops must be evaluated to determine the threshold for benefits. *Viola cornuta* is a high value ornamental crop; however, information on how different rates of biochar rates affect productivity and physiology of Viola cultivars in container production is not known. To determine if biochar rates could increase the productivity of Viola, we mixed a peat-based substrate with 10, 25, and 50% (*w*:*w*) hardwood biochar in two studies on four cultivars. Without fertilizers, 10 and 25% biochar improved plant biomass, growth, root length, and flowering, but 50% biochar was found to have negative effects on plant growth and flowering. Cultivars varied in their response to biochar rates. When fertilizer was applied in the second experiment, biochar rates did not impact growth parameters or flowering. These results suggest that up to 25% biochar can be used in Viola production without detrimental impacts. However, 50% biochar can be used with the addition of fertilizer without negatively affecting plant growth. Biochar can have a short-term impact on the growth characteristics of Viola plants in container production, but fertilization and growing period of Viola may influence these effects. These results indicate that biochar could be the suitable replacement for peat moss, with up to 50% biochar rate in Viola production reducing the environmental and economic burden for peat moss.

## 1. Introduction

Peat moss is one of the main components of soilless potting mixes used in container production in commercial greenhouse industries. Horticultural crops use around 11 million kilograms of peat moss annually [1]. Due to adverse environmental pressure and high rates of peat extraction, alternatives for peat moss are needed [2]. Most of the peat is extracted in Canada and transported to the US, increasing the shipping and handling cost [3,4]. Therefore, different organic and inorganic soil additives are now being substituted for portions of this growing media [5,6]. Biochar is being increasingly explored in production due to its many beneficial properties [5,7].

Biochar is a charcoal-like carbon rich substance obtained from pyrolysis of plant material or organic waste in an anoxic environment [8,9,10]. The particle size, quality, and toxicity of biochar differs by the methods used, substrate, and temperature applied during the manufacturing process [11,12,13]. Because of biochar’s high porosity and surface/volume ratio, it can retain high amounts of exchange cations, resulting in higher adsorption to the planting medium and better productivity of crops [14,15]. As a result, biochar has been shown to increase plant productivity and reduce nutrient leaching [16,17]. The addition of biochar to peat-based media increases porosity in substrates, as well as cation exchange capacity (CEC), pH, and potassium [18,19]. The average price of biochar is almost half as the price of peat moss [20]. Biochar helps to lower the economic cost used to produce horticultural crops in nursery industries by replacing the peat moss up to certain levels [20]. The production cost of biochar can also be lowered if it can be produced onsite with local resources and avoid long distance transportation [21].

Biochar has been found to have positive, neutral, and negative effects on plant growth and yield depending on crop and biochar type [22]. Biochars are mainly classified as hardwood and softwood biochar based on their parent material used [23]. Generally, softwood biochar has lower pH than hardwood biochar [23,24], but it also depends upon the pyrolysis temperature and type of parent material used. Hardwood biochar is mainly used as amendment in greenhouse pot cultivation as it has many of the desired physical and chemical properties required. Hardwood biochar usage is more prevalent in greenhouse horticultural crops in as well as field production [20,21,24,25]. In a study conducted by Ali and Mjeed [9], Chrysanthemum plants grown with eucalyptus biochar had increased plant height, fresh weight, and more buds and flowers. In other studies [26], wood-derived biochar addition resulted in increased plant growth and productivity by increasing plant height in tomato, and increasing leaf area in both tomato and pepper. Reibe et al. [27] also found that spring wheat grown with maize biochar altered plant morphological characteristics by increasing shoot biomass and thickening roots. Alternatively, multiple studies have shown that biochar can have no effect or negative effects on the growth parameters of plants [19]. For example, Schmidt et al. [28] found that the application of larger amounts of wood biochar did not have any significant effect on plant growth parameters of grapevines. In another study by Furtado et al. [29], poultry biochar reduced the growth of sunflowers. These conflicting results illustrate that research must be tailored to specific crops and rates of application to determine the overall impacts on plant production.

*Viola* species are one of the most popular bedding ornamental plants produced in the U.S. [30]. In recent years (2015–2018), sales of Viola have increased, and at the same time, the number of Viola producers have also increased [31]. Violas are annuals or short-lived perennials that grow 10–15 cm tall and produce 2.5 cm flowers [32]. *Viola cornuta* species are grown in full sun to partial shade in USDA plant hardiness zones from 6–11a [33]. They can tolerate acid and neutral soil pH and well-drained, moist soils, but tend to favor more acidic soils [33]. Yet, it should be noted that these values vary according to cultivar, and many breeding lines have been developed to improve heat, disease, and environmental tolerance [34]. Due to small seed size and labor intensive seed production, Violas are often sold and bought as plugs in flats [35,36]. Producing bedding plants in pots or flats for wholesale requires large amounts of peat each year [37]. Peat moss is extensively used in the production of different ornamental plants because of its different physical properties such as high porosity and water holding capacity as well as its high cation exchange capacity [38]. However, mining peat moss has caused the degradation of natural habitats and biodiversity in the areas in which it is mined [39]. Replacing peat moss with biochar at different ratios is an alternative to reduce the over-mining of peat moss but has potential problems [38]. Biochar has been used in ratios of up to 100% (biochar: peat moss) with similar production to 100% peat moss [18], but the results depend upon the variety of plants and the biochar used. As *Viola* species are one of the most popular bedding plants produced, they require a large amount of potting soil in their production, and therefore could be a viable application where biochar could be used to conserve peat moss. However, there is not enough information on how Viola cultivars respond to applications of high percentages of biochar.

We hypothesized that the addition of higher rates of biochar could improve growth and flower production of Viola up to a certain threshold. The objective of this study was to determine the threshold rates at which hardwood biochar can be added to potting soil in container production of Violas without having detrimental effects. To address this, biochar was applied at a low, medium, and high rates, and Viola’s physiological and growth parameters were assessed in two experiments. The interactions between cultivar and biochar rates were evaluated to further determine if there were cultivar specific responses to these application rates. The findings of this research will help ornamental flower producers to determine optimal biochar rates in container production and therefore reduce the amount of peat moss used.

## 2. Results

### 2.1. Growth Parameters

**Figure 1:**
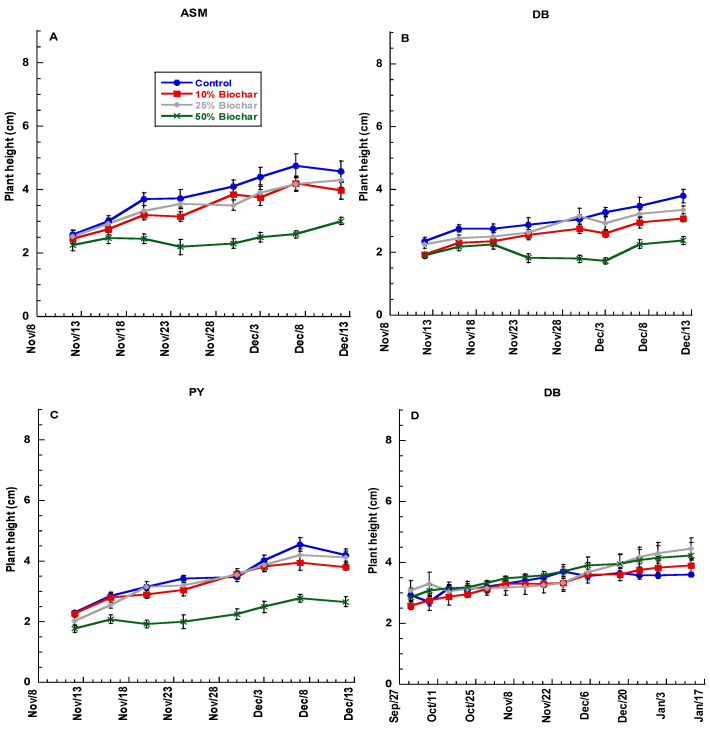

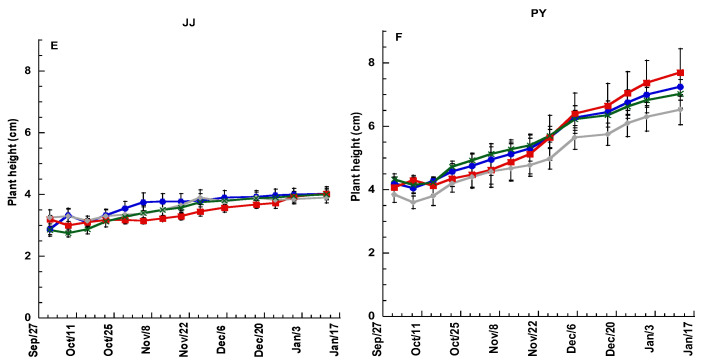
Plant height of *Viola* spp. subjected to rates of 0, 10, 25, and 50% biochar (*w/w*) over time for experiment 1: (**A**) All-season Mix (ASM), (**B**) Deep Blue (DB), (**C**) Penny Yellow (PY). Experiment 2: (**D**) Johnny Jump Up (JJ), (**E**) Deep Blue (DB), and (**F**) Penny Yellow (PY). Bars indicate ± 1 standard error of the mean.

Plant height is an important physiological measurement that indicates plant responses to stress and overall health. Violas are typically selected for compact form, but stunting can be a serious issue if plants are stressed. Plant growth over time varied by experiment, cultivar, and biochar rate, as shown in Figure 1A–F. A repeated measures test on data from experiment 1 showed that both biochar rate and cultivar significantly affected growth over time (*p* ≤ 0.0001 and 0.0001, respectively). In experiment 2, plant height was only significantly affected by cultivars over time (*p* ≤ 0.0001). Biochar rate showed the most dramatic effect on plant height within each cultivar, with plants in the 50% biochar treatments growing the least throughout the first experiment. Plant heights were similar in cultivars initially but were reduced in 50% biochar treatments after one week of growth. In experiment 1, biochar treatments showed an effect on plant growth over time, while in experiment 2, plants showed similar growth over time, regardless of biochar treatment. In experiment 2, growth varied by cultivar only. In the case of PY in experiment 2 (Figure 1F), the plants attained maximum height after application of fertilizer. Furthermore, 50% biochar did not have negative effects on Viola growth during this second experiment.

**Figure 2:**
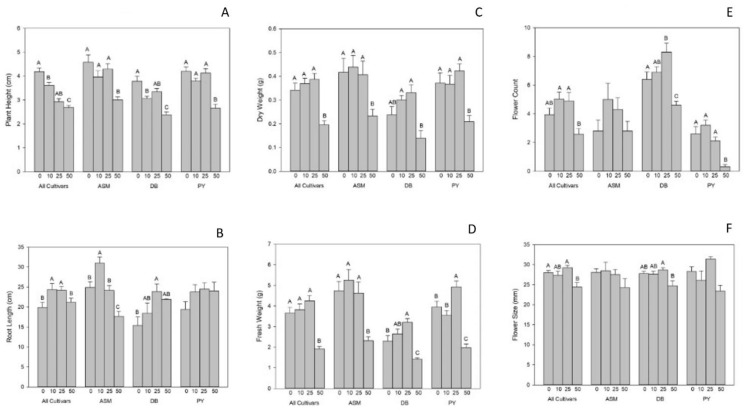
Growth parameters for Violas grown in experiment 1, as separated by cultivar and biochar rate. (**A**) Plant height, (**B**) root length, (**C**) dry weight, (**D**) fresh weight, (**E**) flower count, and (**F**) flower size. Uppercase letters indicate significant differences between biochar rates in experiment 1. Bars indicate ± 1 standard error of the mean.

**Figure 3:**
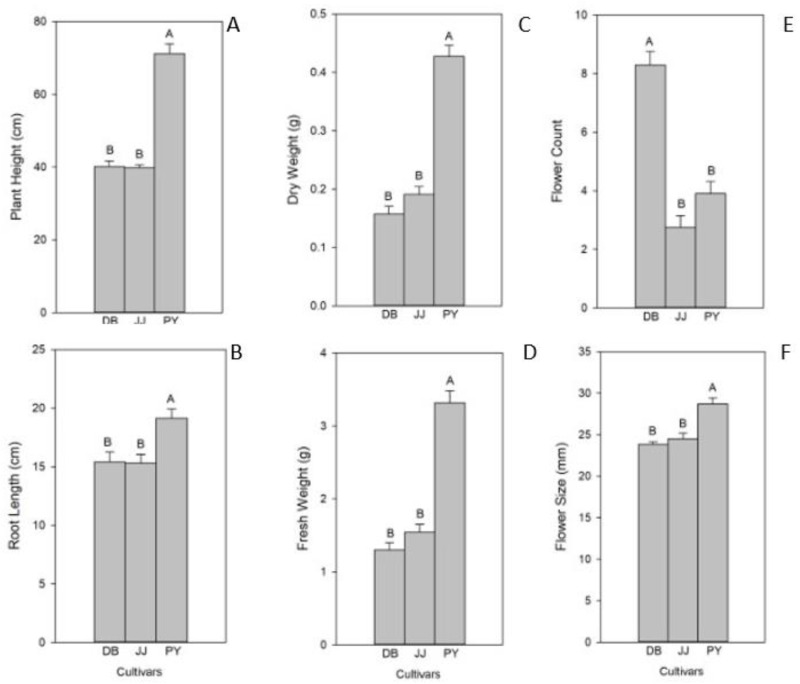
Growth parameters for different Viola cultivars grown in experiment 2. (**A**) Plant height, (**B**) root length, (**C**) dry weight, (**D**) fresh weight, (**E**) flower count, and (**F**) flower size. Uppercase letters indicate significant differences between cultivars in experiment 2. Bars indicate ± 1 standard error of the mean.

To further explore the impacts of biochar on plant height, we evaluated final plant height of both experiments. In experiment 1, final plant height was significantly affected by biochar rates and cultivars, as shown in Figure 2A (*p* biochar < 0.001; *p* cultivar < 0.001; *p* biochar × cultivar = 0.85), but no interaction effect was found between biochar rate and cultivar. The greatest plant height was observed in cultivars grown with no biochar, followed by plants grown with 10% and 25% biochar rates. In experiment 1, final plant height was significantly decreased by 50% biochar. For cultivars, ASM and PY were significantly taller than DB. In experiment 2, the biochar rate had no significant effects on final plant height (*p* biochar = 0.79). However, cultivars did vary significantly in final plant height, with PY being tallest, followed by DB, and then JJ (Figure 3A).

Root length was also significantly affected by the interaction of cultivar and biochar rate in experiment 1 (*p* biochar × cultivar < 0.004; Figure 2B). ASM plants had the greatest root length at 10% biochar, followed by PY at 25 and 50% biochar and DB at 25% biochar (Figure 2B). Root length also varied by cultivar, with DB having shorter roots than ASM and PY at each biochar rate (except ASM at 50%). However, in experiment 2, biochar rate did not affect the root length, whereas cultivar did. Root length was longest in PY, and then DB and JJ, as shown in Figure 3B.

This pattern was also similar for plant fresh weight. In experiment 1, fresh biomass was significantly affected by biochar rate across each cultivar in Viola plants (*p* biochar × cultivar < 0.015; Figure 2D). ASM treated with 10% biochar was found to have the highest fresh weight, and the lowest fresh weights were found in DB plants treated with 50% biochar. While there was no interaction effect for dry weight in experiment 1 (Figure 2C; *p* biochar × cultivar = 0.82), the lowest dry weights were found in plants grown with 50% biochar. Among cultivars, DB had the lowest dry weight, and no significant difference was found among ASM and PY plants in experiment 1. Alternatively, in the second experiment, cultivars were significantly different in fresh and dry weight, with PY having the greatest fresh and dry weight compared to DB and JJ plants (Figure 3C,D).

### 2.2. Plant Flowering

The number of flowers produced was significantly affected by the interaction of cultivar and biochar rate in Viola plants in experiment 1, as shown in Figure 2E. The greatest number of flowers were observed in DB grown with 25% biochar, followed by DB in the 10% biochar treatment, and then the control. The fewest flowers were found in PY with 50% biochar treatments. Biochar rate alone was found to have a significant effect on Viola flowering, with the most flowers being produced in 25% and 10% biochar treatments, and the fewest flowers were found in the 50% biochar treatment. Cultivars also differed in number of flowers produced, with more flowers found in DB followed by ASM and PY (Figure 3E).

In experiment 1, average flower size was significantly affected only by biochar rate. No significant difference was found between control, 10%, or 25% biochar rates, but 50% biochar reduced the average flower size in all cultivars (Figure 2F). In experiment 2, average flower size was significantly affected by cultivar, as shown in Figure 3F. PY had the largest flowers, followed by JJ and DB cultivars. 

### 2.3. Plant Nutrients

Nutrient content in plant samples were higher in experiment 2 than in experiment 1. Because the fertilizer added in experiment 2 contained N, P, K, Mg, B, Cu, Fe, Mn, and Zn, this is to be expected. Some effects of biochar and cultivar were found in experiment 1 (Table 1). N was higher in PY plants with no biochar addition, but there were few significant differences between the controls and biochar treatments overall. Biochar alone affected P, Mg, S, B, Zn, and Mn in experiment 1, with higher rates of biochar resulting in lower nutrient values. In experiment 2, we also saw an interaction effect of biochar and cultivar on N, P, K, Zn, and Fe values (Table 1). However, significant differences in N were only seen between DB at 25% biochar, PY control, JJ, and PY at 50% biochar rates. Significant differences in P, K, Zn, and Fe were similar to those in N. Alternatively, control and 50% biochar treatments had lower concentrations of Mg and Ca. Significantly higher levels of B and Mn were found in the control compared to the 50% biochar treatments. 

## 3. Discussion

There are many factors that could have affected overall plant growth and biomass. The positive effects of biochar seen in the 10 and 25% rates could be due to the positive effects of biochar, such as increased water retention, aeration, and other physical and chemical properties induced by the biochar amendments [40]. Alternatively, the reduction in fresh weight at higher rate of biochar might be due to negative effects of biochar, such as influences on pH or nutrient leaching [26]. This further illustrates that each plant variety and species may have thresholds for positive impacts of biochar when mixed with a substrate. 

The conflicting results presented in experiments 1 and 2 illustrate the impacts that fertilization can have on biochar amended Viola plants. It should be noted that there were some limitations of this study. While the timing was essentially the same each year, and the plugs were from the same source, we did have to grow the plants for a longer time during the second experiment to induce flowering. Fertilization was required due to the extended experimental time, but interesting results were found. Fertilization has been shown to influence plant growth and flowering in other studies [41,42]. While these studies have some conflicting findings, there does seem to be an interaction of biochar and fertilization that impacts plants differently [41,42]. Biochar did impact substrate pH and could have impacted chemical properties that could not be tested due to time and financial constraints. These properties could have influenced toxicity effects, but the evidence presented herein highlights the importance of proper fertility when stressors are present. Fertilization seemed to help plants overcome some of the negative impacts biochar had on development and flowering, which may have aided in plant growth and development. As the results show, there were no significant differences between biochar treatments after application of fertilizers. Therefore, if higher rates of biochar are used, the management of fertilization is highly recommended to overcome any detrimental effects that may be observed. 

### 3.1. Impacts of Biochar on Plant Growth 

Studies indicate there is a threshold at which additional biochar becomes detrimental to plant growth or other parameters [25,43]. While *Viola* species are specifically selected for their compact form, plant height is an indication of stunting and overall physiological effects on plant form. In these experiments, we found conflicting effects of biochar rates on plant height. In experiment 1, we found that 50% biochar significantly reduced the plant height of Viola, while in experiment 2, only cultivar affected final plant height. Researchers studying *Chrysanthemum coronarium* L., *Calathea insignis*, *Solanum lycopersicum* L., and *Tagetes erecta* L. have shown positive effects of biochar in rates ranging from 3–20%, which is less than our two highest rates [9,44,45]. Alternatively, studies conducted by Furtado et al. [29] found that plant height was reduced in sunflower (*Helianthus annus* L.) when poultry biochar rate was increased beyond 5%, and Rajkovich et al. [46] found that the growth of corn was reduced when food waste biochar rate was increased to 7%. This discrepancy between research findings, as highlighted by our experiments, implies that there appear to be multiple factors affecting the interaction between biochar and plant growth. Furthermore, the studies previously mentioned used lower concentrations of added biochar than the highest rate in our experiments, indicating that lower rates have a more beneficial impact in flowering plants. Higher rates of biochar can affect soil pH and other media properties because some biochar are alkaline in nature [40]. In these experiments, pH at the highest rate (50% biochar) was approximately 6.96 and 7.06 for experiments 1 and 2, respectively. In container production, the ideal pH is between 5.5–6.5, above or below which, nutrients are either immobile or bound to soil. Pansies grow well at lower pH, around 5.5 [47]. While this may have contributed to stunted plants at the 50% biochar rate, the conflicting results shown in experiment 2 imply that pH is not likely the cause in these studies. The addition of fertilizer in experiment 2 has overcome the negative effect of biochar at 50% biochar rate, suggesting that only biochar is not enough for nutritional requirement of the Viola. Another significant finding in both experiments was the variation in height between cultivars. Many ornamental plant cultivars vary significantly in growth habits within the same species [48]. Here, PY plants were consistently taller than DB in both experiments. However, this is a cultivar characteristic and is expected.

Biochar has also been shown to affect root length in different plant species. Research conducted by Fascella et al. [49] showed that the addition of 25% conifer biochar produces a similar root length in *Rosa rugosa* to in the control, but root length was decreased when the biochar rate was increased beyond 25%. Similarly, Zhang et al. [44] found that the root length of *Calathea insignis* was significantly increased when 20% coir biochar was added to media, but decreased with 35% biochar. In our first experiment, root length was affected by the different biochar rates, which varied by cultivar. Interestingly, the 10 and 25% biochar rates increased root growth compared to the control in all cultivars. However, the results of our second experiment showed that, while cultivar affected root length, biochar rates did not have significant effects on the root length of Viola. The fertilizer applied in the second experiment provided adequate nutritional requirement for the Viola plants, as plants with 50% biochar in second experiment had higher nitrogen and phosphorous contents (Table 1). These differences could be attributed to cultivar characteristics, where different cultivars of the same species have different growth characteristics in pot experiments or was due to the effects of fertilization mentioned previously [48,50].

Similar patterns were found in the fresh and dry biomass of Viola plants, which were significantly reduced by the addition of 50% biochar in experiment 1. In experiment 2, only cultivar affected plant biomass. 

### 3.2. Impacts of Biochar on Plant Flowering and Size

Previous studies have shown that biochar has variable effects on flower production and size. Conversa et al., [41] found that the flower number of Pelargonium (*Pelargonium zonale* L.) was higher in 30% biochar treatments but was reduced with 70% biochar. In experiment 1, the flowering of Viola followed a similar trend to the results discussed above, where flowering was significantly reduced in 50% biochar treatments and increased at the 10 and 25% rates compared to the control. Researchers have previously found that phytotoxicity can develop from higher rates of biochar and can affect flowering and growth [26]. Therefore, the detrimental effects seen in flowering at the 50% biochar rate could be affected by phytotoxicity. At the same time, the pH of the 50% biochar treatment was approximately 7, which has also been shown to affect flowering [51]. Furthermore, a study by Fornes and Belda [52] found that forest waste biochar rates over 25% significantly decreased the flowering percentage of *Petunia hybrida*, which they partially attributed to pH. However, other studies have shown mixed effects at different rates of biochar. In a pot study performed by Ali and Mjeed [9], the addition of 2 and 3% eucalyptus biochar significantly increased the number of flowers as well as flower size in *Chrysanthemum coronarium* L. Additionally, in a study performed by Alvarez et al. [53], the flower number of Petunia was significantly affected by more fertile substrates, such as vermicompost mixed with biochar. These findings also indicate that biochar can be used as a substrate to induce plant flowering factors. Of the few studies that have been conducted to evaluate the influence of biochar on flower size, Ali and Mjeed [9] found that the diameter of Chrysanthemums was increased at rates of 3% biochar. However, we hypothesized that, as plant growth was negatively impacted by high biochar rates, flower size would also be impacted. In experiment 1, flower size was increased at 25% biochar rates, but this was primarily seen in the DB cultivars. We also found that biochar did not significantly affect flowering number or flower size in experiment 2, indicating that fertilization could overcome the negative consequences of high rates of biochar. A consistent finding throughout our study was that cultivar significantly affected flower number as well as flower size. DB cultivars started flowering earlier and more frequently than PY and JJ cultivars, which resulted in DB having more flowers by the end of the experiment. Conversely, PY flowers were visually larger than the other cultivars. However, it is likely that the primary reason for these differences could be attributed to the larger overall size of PY plants and specific cultivar characteristics that were selected when breeding.

### 3.3. Impacts of Biochar on Plant Nutrient Content

Nutrient deficiency thresholds for each plant may vary based on the cultivar, genotype, and growing condition. For bedding plants, N is generally considered optimum when it is around 3% of the plant dry weight and considered in deficit if lower than 2.75% [54]. According to this, our plants in experiment 1 were deficient in N, whereas plants in experiment 2 were within the range in all but one treatment (PY 50% biochar). It should be noted that drastic deficiency symptoms were not observed in experiment 1 at the time of harvest. However, it is likely that they may have occurred if the experiment had not ended. Thresholds for K deficiency have also been reported as 1–2% [54]. In our experiments, higher K values were found in experiment 1 compared to experiment 2. While there were interaction effects in experiment 2, all values remained above the reported deficiency threshold. The lower K values seen in experiment 2 may have been due to leaching of K, as it has been previously reported that K is easily leached from biochar [55]. It has been found that Violas grown in higher pH soil tend to have B deficiency due to lower availability in the substrate as well as heavy feeding by violas [56]. The pH of substrate in these experiments were slightly higher than recommended for Viola but were less than 7 in most treatments and within optimal ranges for nutrient availability. According to Pitchay [57], Violas show deficiencies in B at approximately 10 ppm, indicating that the plants in both experiments were not B deficient. The differences in nutrient content in the two experiments is likely due to fertilizer application, nutrient leaching by biochar, plant age, and the length of time plants were grown (5 weeks for experiment 1 and 13 weeks for experiment 2). Overall, these results show that biochar may have had some effect on nutrient availability and uptake in container grown plants and further research is needed to determine the effect of biochar on macro- and micronutrients of container grown plants. 

## 4. Materials and Methods

### 4.1. Growing Conditions and Planting Materials

Two pot experiments, from 1 November 2019 to 12 December 2019 (expt. 1) and 10 September 2020 to 12 January 2021 (expt. 2), were conducted in the Horticulture Gardens and Greenhouse Complex of Texas Tech University, Lubbock, TX. Growing media were prepared by mixing commercial potting mixture (Sungro Horticulture, Agawam, MA, USA) with no added fertilizer and three designated rates of dry hardwood biochar (Wakefield Biochar, Columbia, MO, USA) in ratios of 90:10, 75:25, and 50:50 (%wt. of potting mixture: %wt. of biochar). The hardwood biochar was made from oak and composed of 82% organic matter, 63% carbon, and 17.9% total ash, with a pH of 8.6 [24]. The control consisted of only commercial potting mix. The fertilizer was not applied in experiment 1. To treat visible nutrient deficiencies, fertilizer was applied in the second experiment after 7 weeks. In experiment 2, 20 mL of water-soluble Jack’s fertilizer (20N:20P:20K; JR Peters Inc., Allentown, PA, USA) was applied weekly at a 0.50 g/L H_2_O rate, starting seven weeks after planting. EC and pH measurements were taken from 3 containers per treatment using the 1:2 method [58], where two parts water were added to one part soil prior to measurement. The average greenhouse air temperature was 30 °C/20 °C (day/night) for both experiments. Soil pH and EC were measured at the beginning and the end of the experiment (Table 2).

Seedling plugs of Viola were obtained from Desert Rose Plant Farm (Lubbock, TX, USA) and sourced from Eason Horticultural Resources (Covington, KY, USA) for both experiments. In each experiment, three different cultivars of *Viola cornuta* were selected based on availability and varying flower color (Deep Blue (DB), Penny Yellow (PY), and All-Season Mix (ASM)/Johnny Jump Up (JJ)). Two Viola cultivars, DB and PY, and one mixed tray of ASM cultivar, respectively, were used in the first pot study, whereas ASM was replaced with JJ in the second study due to availability constraints. 

Viola seedlings were transplanted into 10 × 10 cm^2^ pots filled with potting soil and specified biochar rates, as described previously. Each treatment was replicated ten times in the first experiment and twelve times in the second experiment for a total of 120 and 144 plants in completely randomized designs. Each pot was irrigated with reverse osmosis city water two to three times per week, as needed.

### 4.2. Growth Parameters

To determine if plant form was affected by biochar treatment, plant height was measured from the base of the plant (soil surface) to the tip of the plant weekly using calipers. Flowering number and flower sizes were measured each week as flowering progressed. Flower size was measured at the widest point of each fully expanded flower. At the end of the experiments, plants were harvested, roots were cleaned, and root length, fresh biomass, and dry biomass were measured. 

### 4.3. Plant Nutrient Analysis

Oven-dried plant samples (264 plants total) were sent to Waters Agricultural Laboratories (Camilla, GA, USA) for the determination of macro- and micronutrient content in Viola plants.

### 4.4. Statistical Analysis 

The experiments used a completely randomized design, with biochar and cultivar as different treatment levels. A manova repeated measures test (JMP Pro 15.0.0, SAS Inc., Cary, NC, USA) was used to determine significant differences over time. Analysis of variance (ANOVA) was carried out between biochar rates and cultivars to determine differences and interactions between treatments and cultivars. ANOVA tests were also conducted within cultivar if significance was identified. Significance levels were set at 0.05. When differences were significant, Tukey’s HSD test was used to establish a significant difference among means. A correlation matrix was used to determine relationships between plant growth measurements with flowering of Viola. 

## 5. Conclusions

We found that biochar can positively influence the physiology of Viola in short-term production, whereas the negative stress of higher biochar rates can be minimized by the application of fertilizer in the long term. This study also suggests that the addition of hardwood biochar (by weight) in potting media can be beneficial and economical for Viola. Biochar can be a good substitute source for peat moss in horticultural industries, reducing the environmental issues related to high usage of peat moss. This research yields promising insight into how production practices and soilless media can influence the growth of Viola cultivars in greenhouse production. However, further research is necessary to fully understand the interaction between biochar and growth parameters of Viola.

## Figures and Tables

**Table 1:** Plant nutrient analysis for macro and micro nutrients in Viola cultivars (All-season mix (ASM), Deep Blue (DB), Penny Yellow (PY), and Johnny JumpUp (JJ)) treated with different biochar rates.

Cultivar (C)	Biochar Rate (B)	N (%)	P (%)	K (%)	Mg (%)	Ca (%)	S (%)	B (ppm)	Zn (ppm)	Mn (ppm)	Fe (ppm)	Cu (ppm)
Experiment 1												
ASM	Control	1.90 bcd	0.43 A	3.63	0.83 A	1.26 abcde	0.19 B	38.00 A	107.33 A	206.00 A	630.00	8.67
	10%	2.08 abcd	0.38 A	3.63	0.79 AB	1.18 bcde	0.21 A	30.00 B	96.67 A	138.33 B	329.33	8.67
	25%	1.91 bcd	0.39 AB	3.6	0.71 BC	1.29 abcde	0.19 AB	31.67 B	91.67 B	103.67 C	245.33	7.67
	50%	1.58 d	0.32 B	3.48	0.74 C	1.06 de	0.16 AB	27.67 B	78.33 B	151.33 B	289.00	7.33
DB	Control	1.94 bcd	0.35 A	3.78	0.93 A	1.49 abc	0.19 B	28.33 A	95.00 A	225.00 A	635.00	8.00
	10%	2.21 abc	0.36 A	3.74	0.94 AB	1.67 a	0.22 A	24.33 B	104.33 A	226.67 B	639.33	8.33
	25%	2.11 abc	0.37 AB	3.61	0.81 BC	1.62 b	0.20 AB	22.67 B	71.33 B	132.33 C	382.00	8.33
	50%	1.78 cd	0.30 B	3.64	0.82 C	1.43 abcd	0.18 AB	24.67 B	75.00 B	181.00 B	363.33	7.33
PY	Control	2.49 a	0.57 A	2.86	0.92 A	1.53 ab	0.13 B	35.33 A	147.00 A	331.67 A	627.67	8.67
	10%	2.31 ab	0.49 A	3.39	0.85 AB	1.27 abcde	0.16 A	32.00 B	136.33 A	205.67 B	544.33	7.67
	25%	1.89 bcd	0.37 AB	3.68	0.67 BC	1.11 cde	0.18 AB	28.33 B	87.33 B	106.00 C	341.00	7.33
	50%	1.8 bcd	0.32 B	3.78	0.75 C	0.99 e	0.17 AB	26.67 B	99.33 B	228.67 B	292.33	9.67
F-ratio		5.97	3.20	1.52	2.92	7.66	4.46	6.81	6.20	5.58	0.86	1.26
df		11,24	11,24	11,24	11,24	11,24	11,24	11,24	11,24	11,24	11,24	11,24
P C		0.0065	0.0242	0.2110	0.0147	<0.0001	0.0003	<0.0001	0.0002	0.0067	0.5557	0.7469
P B		<0.0001	0.0047	0.5553	0.0023	0.0026	0.0144	0.0001	<0.0001	<0.0001	0.0920	0.6413
P B × C		0.0374	0.1858	0.1278	0.8520	0.0260	0.0759	0.3441	0.1884	0.1745	0.9835	0.1165
Experiment 2												
JJ	Control	3.35 abc	0.64 abc	2.24 abc	1.01 B	1.57 B	0.07 b	36.00 A	228.00 ab	359.67 A	263.00 bc	10.67
	10%	3.32 abc	0.71 abc	1.80 bc	1.11 A	1.67 A	0.07 b	31.00 AB	253.33 a	289.00 AB	286.67 abc	10.67
	25%	2.90 abcd	0.56 bc	1.73 bc	0.98 AB	1.58 AB	0.06 b	31.00 B	191.33 bc	249.00 BC	221.00 c	9.67
	50%	2.75 bcd	0.51 bc	2.50 ab	0.95 B	1.41 AB	0.10 b	30.67 B	167.33 cde	224.67 C	487.67 ab	10.00
DB	Control	3.07 abcd	0.62 abc	1.76 bc	1.04 B	1.94 B	0.06 b	29.00 A	151.00 cde	226.33 A	304.33 abc	10.00
	10%	3.37 ab	0.72 abc	1.73 bc	1.09 A	2.28 A	0.07 b	27.33 AB	157.33 cde	209.67 AB	303.67 abc	11.00
	25%	3.64 a	0.87 a	1.82 bc	1.03 AB	2.1 AB	0.06 b	23.67 B	146.00 cde	141.00 BC	253.33 bc	12.67
	50%	3.09 abcd	0.59 abc	1.41 c	1.03 B	2.07 AB	0.07 b	21.33 B	130.33 de	138.00 C	392.67 abc	16.33
PY	Control	2.36 d	0.45 c	3.03 a	0.83 B	1.38 B	0.15 a	31.67 A	123.00 e	172.00 A	539.00 a	8.33
	10%	3.00 abcd	0.65 abc	2.28 ab	0.98 A	1.68 A	0.08 b	31.00 AB	182.67 bcd	188.00 AB	380.33 abc	9.00
	25%	3.03 abcd	0.75 ab	1.86 bc	0.96 AB	1.72 AB	0.06 b	31.67 B	179.00 bcde	152.67 BC	281.67 abc	8.67
	50%	2.57 cd	0.52 bc	2.08 bc	0.84 B	1.61 AB	0.06 b	29.00 B	130.33 de	131.00 C	239.67 bc	9.00
F-ratio		5.53	4.37	6.81	4.51	10.86	7.81	7.23	12.48	7.63	3.86	1.96
df		11,24	11,24	11,24	11,24	11,24	11,24	11,24	11,24	11,24	11,24	11,24
P C		0.0001	0.0266	<0.0001	<0.0001	<0.0001	0.0049	<0.0001	<0.0001	<0.0001	0.3534	0.0088
P B		0.0055	0.0080	0.0042	0.0075	0.0087	0.0073	0.0017	<0.0001	0.0006	0.0270	0.4137
P B × C		0.0232	0.0395	0.0024	0.4671	0.3228	<0.0001	0.2429	0.0055	0.4948	0.0021	0.3631

Means separations were determined via Tukey’s HSD tests. Lowercase letters indicate significant interaction effects between cultivar and biochar rate. Uppercase letters indicate significant differences between biochar rates. P B × C indicates interaction between biochar rates and cultivars.

**Table 2:** Average EC and PH of the growing media at the end of experiment.

	Expt. 1				Expt. 2			
	EC_initial_	pH_initial_	EC_final_	pH_final_	EC_initial_	pH_initial_	EC_final_	pH_final_
Control	0.45 dS/m	6.32	1.40 ds/m	6.29	0.47 dS/m	6.28	0.12 dS/m	6.41
Biochar 10%	0.43 dS/m	6.70	1.19 ds/m	6.35	0.42 dS/m	6.63	0.98 dS/m	6.53
Biochar 25%	0.37 dS/m	6.87	1.19 ds/m	6.66	0.39 dS/m	6.81	0.67 dS/m	6.94
Biochar 50%	0.28 dS/m	7.17	1.03 ds/m	6.96	0.29 dS/m	7.10	0.51 dS/m	7.06

## Data Availability

Data are available by contacting the corresponding author.
